# Supplementary material for: B-Cell Lymphomas Secrete Novel Inhibitory Molecules That Disrupt HLA Class II-Mediated CD4+ T-Cell Recognition
Source: Cells. 2025 Aug 7;14(15):1220. doi: 10.3390/cells14151220 (PMC12346740; doi:10.3390/cells14151220)
Supplement: Supplementary file 1 [file cells-14-01220-s001.zip › cells-3786180-supplementary.pdf]

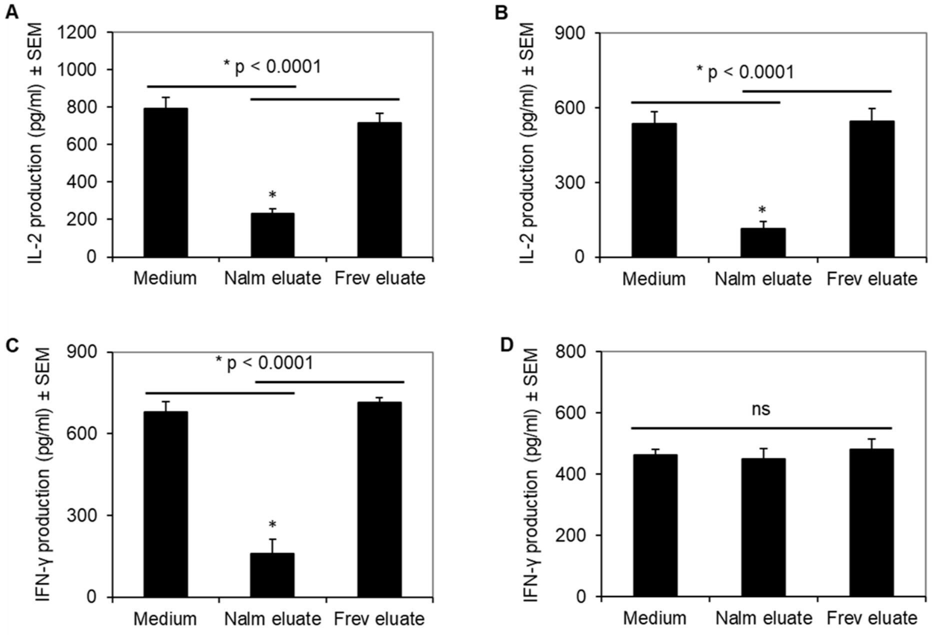

Supplemental Figure S1. Effects of BL-associated inhibitory molecules on Ag presentation by HLA class II and class I molecules. (A) HLA-DR4 and (B) HLA-DR1 expressing B-cells (Frev) were incubated with specific peptides (10μM) plus/minus BL-derived molecules (50 ug/mL), followed by co-culture with peptide-specific T-cell hybridoma lines. T-cell production of IL-2 was quantitated as described in the text. HLA-DR7-positive EBV-transformed B-cells (165.1, 5×10^5) were generated and incubated with BL-derived molecules (50 ug/mL) plus/minus EBNA1(482-496) peptide (10μM) overnight. After incubation, cells were cocultured with peptide-specific CD4+ T-cells. Data suggest that BL-eluted molecules disrupt HLA DR1/DR4/DR7-restricted CD4+ T-cell responses. Assays with HLA-A2-restricted Ag presentation showed that BL-derived molecules were unable to disrupt CD8 cell responses. Briefly, peripheral blood monocytes (PBLs) were stimulated/restimulated with EBNA1 peptide (565-574, FLQTHIFAEV) plus 10 U/mL IL-2 for 7-10 days, washed, and cocultured with the T2 cell line in the presence or absence of BL-derived molecules (50 ug/mL) for 48 hrs. ELISA was performed to measure IFN-γ in the culture supernatants. Statistical analyses were performed by Student’s t-test. Data are representative of three separate experiments.
